# Overload and neovascularization of shoulder tendons in volleyball players

**DOI:** 10.1186/1756-0500-5-397

**Published:** 2012-08-01

**Authors:** Angela Notarnicola, Francesco Fischetti, Donato Gallone, Lorenzo Moretti, Pasquale Pignataro, Silvio Tafuri, Biagio Moretti

**Affiliations:** 1 Course of Motor and Sports Sciences, Faculty of Medicine and Surgery, University of Study of Bari, Lungomare Starita 1, 70123, Bari, Italy; 2 Orthopedics Section, Department of Neuroscience and Organs of Sense, Faculty of Medicine and Surgery, University of Study of Bari, General Hospital, Piazza Giulio Cesare 11, 70124, Bari, Italy; 3 Department of Biomedical Sciences and Human Oncology, Faculty of Medicine and Surgery, University of Study of Bari, General Hospital, Piazza Giulio Cesare 11, 70124, Bari, Italy; 4 Radiology Section, Faculty of Medicine and Surgery, University of Study of Bari, General Hospital, Piazza Giulio Cesare 11, 70124, Bari, Italy

**Keywords:** Shoulder, Overuse, Volleyball

## Abstract

**Background:**

In overhead sports like volleyball, the onset of a rotator cuff tendinopathy due to functional overload is a common observation. An angiofibroblastic etiopathogenesis has been hypothesized, whereby a greater anaerobic metabolism occurs in critical zones of the tendon with a lower degree of vascularization; this would induce collagen and extracellular matrix degradation, that could then trigger a compensatory neovascularization response. We performed a clinical observational study of 80 elite volleyball players, monitoring the perfusion values of the supraspinatus tendons by oximetry.

**Results:**

No statistically significant differences were found between the oximetry data and age, sex or years of sports activity, nor when comparing the right and left arm or the dominant and non-dominant arm. A statistically significant difference was found for the dominant arm values in relation to the competitive role, higher values being obtained in outside hitters (62.7%) than middle hitters (53.7%) (p = 0.01), opposite hitters (55.5%) (p = 0.02) and libero players (54.4%) (p = 0.008), whereas there were no differences in setters (56.2%) (p > 0.05).

**Conclusions:**

The different tendon vascularization values found in players with different roles in the team may be attributed to a response to the specific biomechanical demands posed by the different overhead throwing roles.

## Background

Volleyball is one of the most popular sports in the world, played by more than 200 million athletes worldwide [1]. It was first introduced in 1895 by an American physical training teacher, William G. Morgan, as a recreational activity for students at college in the USA, and its popularity spread rapidly. It became an summer Olympic games sport in 1964 [2]*.* It is a non contact sport, since the players in the opposite teams are separated by a net, so the incidence of injury is relatively low. However, it demands rapid movements to change the body position in space, in the horizontal, vertical and rotational directions, and the arms undergo the greatest strain during overhead movements, as they do in other sports activities like basket ball, baseball, tennis and swimming, in which degenerative diseases of the rotator cuffs are also common [3].

**Figure 1:**
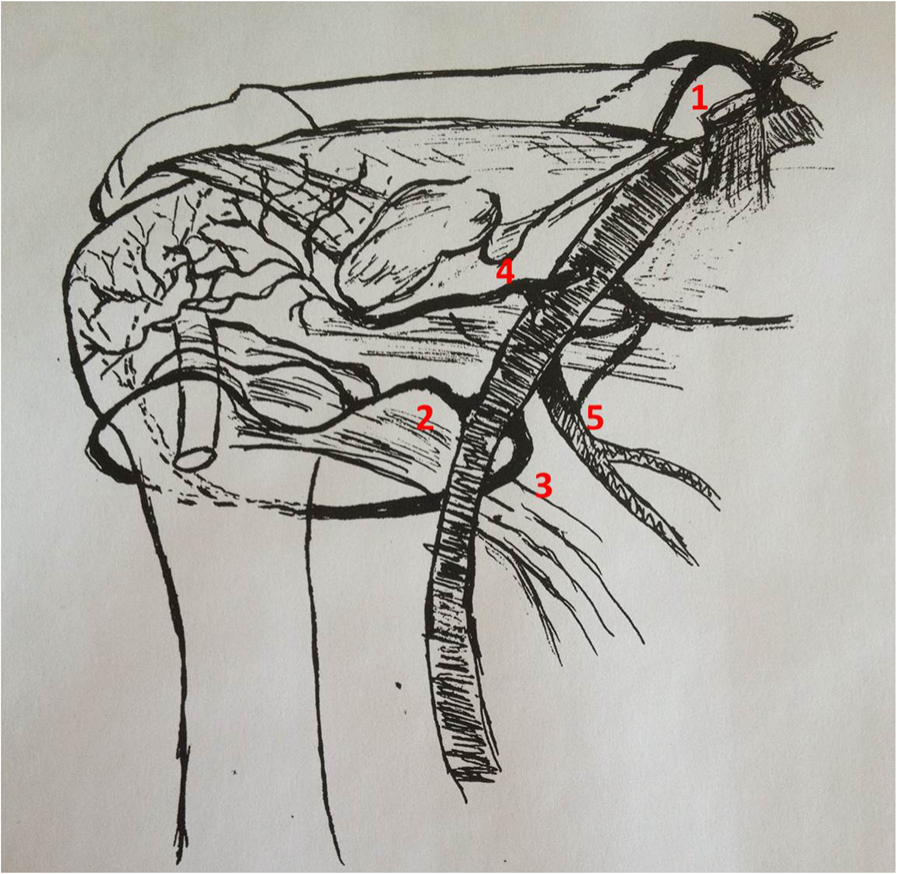
**The axillary and brachial arteries supply the branches of blood vessels to the shoulder.** The branches contributing to the blood supply of the rotator cuff tendons are as follow: 1. Suprascapular artery (vascularizes the infraspinatus and teres minor tendons); 2. Circumflex anterior humeral artery (vascularizes subscapularis tendon); 3. Circumflex posterior humeral artery (vascularizes infraspinatus and teres minor tendons); 4. Thoracoacromial artery (vascularizes supraspinatus tendon); 5. Subscapular artery (minimal contribution to vascularization of the anterior part of the rotator cuff tendons).

Acting as dynamic stabilizers of the scapolohumeral joint, the rotator cuff tendons (RC) are under continual strain in sports involving overhead throwing, as they maintain the humeral head centered within the glenoid cavity. Because the tendons are continually sliding around the subacromial space, they can undergo a degenerative response, developing inflammation, thinning and lesions [4]. In overhead throwing, the posterior portion of the shoulder is frequently found to suffer from a greater rigidity, manifesting in the form of a limitation of internal rotation movements [5]. This can lead to an antero-superior shift of the humeral head that will contribute to a further evolution of the RC tendinopathy [6]. Overhead athletes have a higher risk for shoulder region tendinopathy [7-9]. In the literature little research has yet been conducted on “borderline” pathophysiological alterations, before the degenerative process has become manifest [10,11]. The major arterial supply to the rotator cuff is derived from the ascending branch of the anterior humeral circumflex artery, the acromial branch of the thoracoacromial artery, as well as the suprascapular and posterior humeral circumflex arteries [12] (Figure 1). It has been suggested, in view of the fact that the tendon disease is most frequently localized in the critical area where there is a lesser degree of vascularization, that insufficient perfusion during functional overload could be responsible for the tendon degeneration [6,13-15]. Hypoxia would cause a greater concentration of lactic acid in the interstitial fluid, as demonstrated by micro-dialysis tests made in patients with chronic tendinosis as compared with subjects with healthy tendons. This would indicate that, due to overuse of these tendons, the greater anaerobic metabolism induces a process of collagen and extracellular matrix degradation, which would in turn trigger a compensatory neovascularization response [16]*.* Vascular endothelial growth factor (VEGF), a powerful angiogenic cytokine, plays an important role in this context by regulating the response to hypoxia and inflammation during pathological soft tissue processes [17].

To study the pathophysiology of rotator cuff overuse tendinopathies in overhead sports, we conducted a clinical study of a group of elite volleyball players. The oxygenation values of RC tendon tissue perfusion were recorded, to assess the situation in states of tendon overuse. The rationale for the study was to verify a correlation between vascularization differences of shoulder tendons and different roles in the team. This finding could contribute to develop a tool for evaluating different degrees of exposure to the risk of eventually developing degenerative tendon disease.

**Figure 2:**
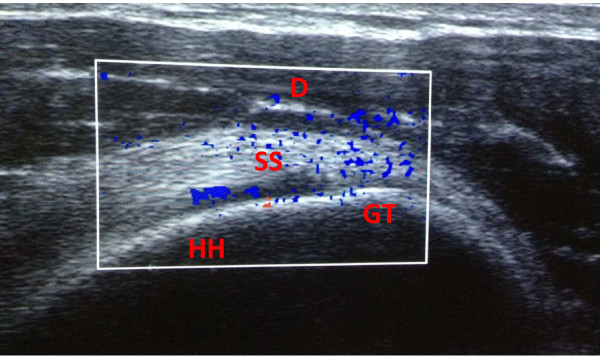
**Power Doppler US imaging shows vessels mainly at supraspinatus tendon during examination of volleyball player’s shoulder.** Supraspinatus tendon (SS), greater tuberosity (GT), humeral head (HH) and deltoid muscle (D) are labeled. The oximetry value was 63%.

In our preliminary experience Power Doppler US images of shoulder obtained in matched control cases showed supraspinatus tendon vessels (Figure 2), without evident differences between examined subjects. In this clinical model, we hypothesized that oximetry could indirectly reveal even modest increases in the local microcirculation that are not detectable by power-Doppler [18]. Power-Doppler can assess high velocity flows, especially in the large vessels, but is less efficient in the study of low velocity flows in the microcirculation and the relative regional perfusion [19,20]. As Eriksson and colleagues [20] point out, Doppler examinations are designed to assess the mean or maximal velocity in a vessel rather than “inflow” to a tissue. For this reason, therefore, we adopted oximetry as the assessment method since it may be more sensitive to alterations in the microcirculation [21,22].

The purpose of the study is to evaluate the influence of age, gender, dominant arm, role and number of years activity on rotator cuff tendon strain, with the aim of developing strategies to improve peripheral perfusion in volleyball players. This study was designed to test the hypothesis that there are variations in tendon vascularity even among players of the same sport, that can be assessed and quantified by oximetry.

## Methods

We set up a clinical observational study designed to recruit 80 volunteer elite volleyball players in professional teams to undergo oximetry tests, halfway through the tournament season. The study was approved by the local Ethics Committee of Bari University General Hospital and all subjects underwent a prior interview and complete illustration of the study aims and design, at the end of which they signed informed consent to take part.

Only players with no pain in the shoulder were eligible to enter the study, and exclusion criteria included any sign at clinical examination of a disorder of the scapular girdle (painful acromion, coracoid process, acromioclavicular joint or sternoclavicular joint [23], cross-body adduction test [24] and Paxinos sign [25]) an incomplete active range of movement (a range of motion of 160°–180° in abduction/30°–40° in adduction, 160°–180° in flexion/30°–45° in extension, 90°–100° in internal rotation/70°–80° in external rotation was needed to enter the study) as well as any pain or weakness shown by the main specific clinical tests used to assess the integrity of the RC tendons [26]. A 40 cm, 360° goniometer was used, marked in 1° increments, with two adjustable overlapping arms [27]. Abduction and adduction measurements were made by placing the stationary arm parallel to the chest and moving the other arm outward or in toward the chest. Flexion and extension measurements were obtained with the shoulder at 0 degrees of abduction and moving the arm forward and backward. Internal and external rotation were assessed starting with the arm lying parallel to the chest and the elbow at 90° flexion, then moving the arm inward and outward. All data were collected by the same physician (AN), who has 7 years of clinical and teaching experience in musculoskeletal disorders and shoulder rehabilitation.

Assessments of the rotator cuff included clinical tests taken from standard texts [28]: the Jobe test [29] infraspinosus test [30], Lift-off test [31], external rotation lag sign [32], Neer impingement test [33] and Hawkins impingement test [34]. Individual characteristics recorded were age, sex, as well as dominant arm, the number of years of sports activities and the specific role played in the team.

Inclusion criteria were professional volleyball activities for at least 5 years, a minimum frequency of 3 training sessions per week, age range between 12 and 40 years. Exclusion criteria were previous surgery, intra- or peri-articular infiltrations in the last 6 months, a clinical/instrumental diagnosis of RC tendinopathy in course or in the clinical history.

The study consisted of monitoring the tissue perfusion values in both shoulders by oximetry. Subjects were asked to hold their arms hanging down their sides for half an hour before starting a training match, and then the oximetric values of both the dominant and the contralateral arm were measured using the cerebral and somatic/peripheral oxymeter INVOS 5100 C (Somanetics Corporation, USA), approved by the Food and Drug Administration (FDA). This is employed to monitor adult and pediatric patients, newborns and premature infants when their clinical conditions expose the brain or a body part to the risk of hypoperfusion or ischemia, such as in heart or vascular surgery, in interventional cardiac catheterization laboratories, intensive postoperative care and neonatal and pediatric intensive care units. The systems exploits the principle of Near-infrared spectroscopy (NIRS) and is equipped with the SomaSensorR and NIRSensorTM and a LED photo-emitter that emits a light source with a wavelength ranging between 650 nm and 1100 nm, as well as a double photo-probe that records the different levels of hemoglobin absorption according to whether the tissue is well oxygenated or not. It thus enables invasive assessment of the level of tissue oxygen saturation (cerebral or somatic) and indirect evaluation of the regional perfusion [34].

**Figure 3:**
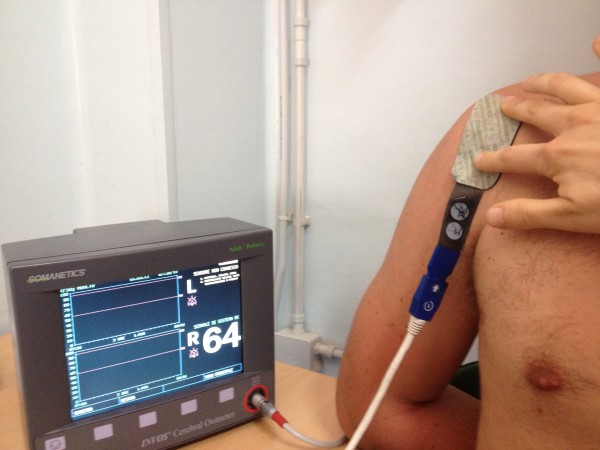
Procedure for placing the oximetry sensor on the volleyball player’s shoulder to assess the tissue perfusion values.

The sensor was placed under ultrasound guidance at the level of the supraspinatus insertion on the humeral trochitis with the arm in internal rotation and adduction, and could evaluate a depth of 2 to 4 cm over a surface of 2 cm^2^ (Figure 3).

The data were collected in a dedicated database created with File Maker Pro software and analysed with STATA MP11 for Mac (Stata Corp LP 2011) software.

Quantitative variables were expressed as mean, standard deviation and range, and qualitative data as ratios, indicating the 95% confidence intervals.

To compare differences between means, student *t* test for independent samples was employed and, when necessary Wilcoxon and Kruskall Wallis tests; to quantify differences between ratios the chi-square test was applied. Chi-square test was used to evaluate differences between proportions.

The individual characteristics of each volleyball player were related to the oximetry values in the dominant arm. The choice to monitor the dominant arm was made because even if volleyball is a prevalently symmetrical sport, in some moments of the game it is asymmetrical [35,36]*.*

To assess any correlation between age and dominant arm oximetry values, and between the number of years of sports activity and dominant arm oximetry, a linear regression model was applied and the determination coefficient (r2) and Fisher tests were calculated. Significance was set at a value of p < 0.05.

## Results

Among the 80 elite volleyball players making up the study population, there were 64 females (80%; 95% CI = 69.6–88.1) and 16 males (20%; 95% CI = 11.9–30.4). Mean age was 22.8 years (SD = 6.2; range 13–38), being 25.9 years (SD = 5.9) in males and 21.9 years (SD = 6.1) in females (t = 2.33; p = 0.02). The mean number of years of sports activities was 12.3 years (SD = 6.0; range = 5–26), with no statistically significant differences between females (mean = 12.4 years; SD = 6.1; range = 5–26) and males (mean = 11.8; SD = 5.9; range = 5–21; t = 0.35; p = 0.36).

The role of middle hitter was played by 25% (n = 20; 95% CI = 16–35.9), outside hitter by 31.3% (n = 25; 95% CI = 21.3–42.6), libero by 17.5% (n = 14; 95% CI = 9.9–27.6), opposite hitter by 13.8% (n = 11; 95% CI = 7.1–23.3) and setter by 12.5% (n = 10; 95% CI = 6.2–21.8). There were no statistically significant differences in the distribution of the subjects by role in the two sexes (chi-square = 5.4; p = 0.24). The left arm was dominant in 10% of the sample (n = 8; 95% CI = 4.4–18.8).

The mean oximetric values for the dominant arm were 55.9% (SD = 8.8; range = 37–83), with no significant differences between subjects with a dominant right arm (mean = 55.9; SD = 9.0; range = 37–83) or dominant left arm (mean = 56.2; SD = 6.2; range = 49–67; t = 0.10; p = 0.91).

Dividing oximetric values by gender, standard deviation was not homogeneous and so non parametric tests were applied. No statistically significant differences in oximetric values emerged in relation to the variables age (r2 = 0.01; F = 0.39; p = 0.53) and sex (H = 0.9, p = 0.34).

**Figure 4:**
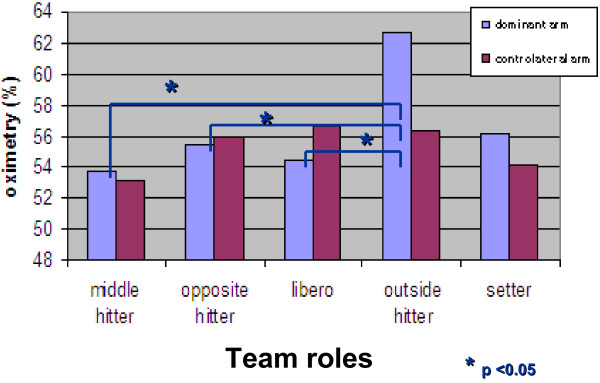
Statistical analysis of the mean dominant arm and contralateral arm oximetry values (expressed as %) in the study population subdivided by team role.

Instead, the mean perfusion values at the dominant arm were significantly different according to the role played in the team. Players with the role of outside hitter had statistically significantly higher values (62.7%) than middle hitters (53.7%) (p = 0.01), opposite hitters (55.5%) (p = 0.02) and libero players (54.4%) (p = 0.008), whereas there were no differences in setters (56.2%) (p > 0.05). By contrast, no statistically significant role emerged as regards the mean perfusion values of the contralateral arm according to the team role (Figure 4).

In the logistic regression model, the number of years of sports activities did not influence the oximetric values of the dominant arm (r2 = 0.01; F = 1.01; p = 0.29).

## Discussion

Although there are biomechanical differences among specific sport actions, since overhead throwing and the spike are required in volleyball, the swing in golf and the serve in tennis, all overhead movements pose a considerable mechanical overload on the glenohumeral joint complex and the rotator cuff tendons. Approximately 70% of overhead ball players have a history of shoulder problems [37]. The knowledge of the natural history of degenerative disorders due to shoulder overuse in overhead athletes has evolved remarkably in the last decade [7-9]. In the last years many studies of the kinetics and cinematics of overhead throwing have been made [38-44]. Using US or MRI, some Authors have demonstrated the presence of tendinopathies and bursitis, and even partial lesions, in overhead athletes with an asymptomatic clinical picture [10,11]. These same Authors stress the fact that at the subsequent follow-up the subjects examined had not developed symptoms [10].

In the present study we probed local tissue perfusion in a population of volunteer elite volleyball players. We started on the assumption that the histopathological picture of tendinopathy has been defined as “angiofibroblastic tendinosis” [44] on the basis of US power-Doppler examinations, that constantly reveal vascular hyperplasia [44,45]. Besides, biopsy shows an increased density of the microcirculation, associated with the proliferation of endothelial and smooth muscle cells, as well as perivascular inflammatory cells, fibroblasts and nociceptive fibres [46-49]. Recent clinical and experimental research suggests a possible pathogenic role of angiogenesis in inducing and maintaining tendon disorders [46,50]*.* VEGF expression is increased in response to mechanical loads and traumatic stimuli, promoting the proliferation, migration and survival of endothelial cells and modulating the permeability of the capillaries in response to local edema [51]. Some Authors [52,53] have demonstrated, in painful shoulders, a prevalently greater perfusion during tendinopathies, featuring pictures ranging from moderate neovascularization (insufficient to show the newly formed vessels at power-Doppler) to gross increases exceeding 50% of extension of the vessels in the tendon tissue. By oximetry in a previous experience in 30 patients affected by RC tendinopathy we demonstrated statistically significantly higher values in the dominant arm than in the unaffected contralateral arm (80.6% vs 45.66%, p < 0.0001). After treatment, a reduction of local perfusion was demonstrated at 2 and 6 months follow-up (53.2% and 51.5%, respectively, p < 0.001), associated with an improved clinical-instrumental picture [54]. On the other hand, the vascular theory for tendinopathy has not yet been accepted by the international Community. Lewis and colleagues demonstrated that neovascularity does occur in subjects with a clinical diagnosis of rotator cuff tendinopathy [55]. However, some authors have suggested that the consequent sclerosing therapy for tendinopathy may not guarantee the promised results [56].

In the literature, the need to identify, classify and define the sports profiles of overhead sports athletes at higher risk of RC overuse has been pointed out [52]*.* In volleyball the basic movement that starts off the game is the serve [2]*.* The ball is received and then set, or it is passed from one to another player by passing. The spike is the one-handed strike or slam given to the ball. The “block” is formed when one or more players raise their arms above the net to stop the adversary’s shot. In volleyball the players may be assigned one of five different roles: middle hitter, libero, outside hitter, setter and (spiker) opposite hitter [2]. The middle hitter is particularly good at the basic movements spike and block. The libero is a specialist in the basic movement of reception of the ball. The outside hitter is not only good at the basic movement of the spike, but must also have a good ball catching technique. The setter has the task of setting the ball for the hitters. The opposite hitter largely adopts the basic movement of the spike, having no ball reception task.

Our oximetric study of RC tendon perfusion showed that volleyball players have progressively increasing values according to their role in the team, passing from setter through middle hitter, libero, opposite hitter, to outside hitter, who has the statistically significantly highest oxygenation values. These different levels of tendon vascularization according to the different player roles could be attributable to the specific biomechanical joint demands made by overhead throwing movements. Previous studies have shown a greater incidence of tendon disorders in outside hitters and setters than other volleyball players [57,58], so we could hypothesize that the increased local microcirculation we found in these two volleyball roles could be the pathophysiological setting for an increased prevalence of degenerative disease. The differences related to the player role were statistically significant only for the dominant arm not the contralateral arm. This finding is in agreement with the assumption that there is asymmetrical shoulder strain in volleyball players [35,36]. An interesting outcome emerged for setter players, who are typically involved in bilateral overhead activities, given the requirements of the position. We expected that this subset of subjects would show increased vascularization bilaterally. Instead, we found a different vascularization in the two arms, with greater perfusion in the dominant arm. In literature it has been reported that in sports requiring symmetrical body movements, such as swimming, running, football or cycling, the dominant arm undergoes a greater muscle-tendon strain [59-61]. This can be explained by the fact that humans have a natural tendency to use one side of the body in preference to the other [62,63]. The tendency to use one side in preference to another originates from both genetic sources and development after birth [64]. Having a preferred side can cause asymmetries, both as a primary cause and as a secondary cause through strength and neural development resulting from favored use and consequent overuse of the dominant side [65-67].

Knowledge of the risk factors is essential in order to develop preventive measures to reduce tendon overuse. An interesting finding in our study is the lack of differences in perfusion according to age under 40 years old and between males and females. In the literature it has been reported that age over 40 years and male sex are risk factors for the onset of tendinopathies. Nevertheless, in overhead sports it has been shown that these variables were not correlated to the onset of a shoulder overuse syndrome [68]*.* The data obtained in the present case series support the hypothesis that under the age of 40 there may be no age or gender-related differences in the perfusion response to tendon rotator cuff overuse in overhead sports. We hypothesize that during the phases of training for matches it may not be necessary to differentiate the type of muscle-tendon strain on the basis of these parameters, in order to reduce the tendon neoangiogenetic response. Additional research is required to improve our understanding of the causative factors modulating tendon perfusion. In the design of our study we did not include blood sampling, whereas it might be interesting, in future research, to see whether blood group may have any influence on the tendon perfusion response, bearing in mind that a correlation between blood group 0 and a greater incidence of tendinopathy has already been demonstrated [69].

Moreover, no difference was found as regards the years of sports activity. Burkart reported that overhead athletes with a painful shoulder often show muscle weakness, suggesting that the symptoms may be produced by overuse of the shoulder girdle, not attributable to the amount of training but secondary to an altered biomechanical situation in this district, induced by the attempt to compensate the lesser muscle power during the first phase of the ball throw [7,8]*.* It has been estimated that volleyball players toss the ball overhead an average of 40,000 times in each tournament season [40]*,* but this could even be an underestimate. In any case, the available data suggest that the onset of a shoulder impingement syndrome could be imputable not only to the number of repetitions of the overhead movements in each season, already remarkable, but also and above all to an imbalance between the eccentric force of the extra-rotator cuff muscles (supraspinatus, infraspinatus and teres minor muscles) and the concentric force of the antagonists, exposing the scapolohumeral girdle to an enormous cumulative overload during spikes, serves etc. [43].

The weak points of the present work include the absence of prospective monitoring and long follow-up, that could follow the course of onset of a disorder over time. Extension of the work to other overhead sports could also provide important verification and support the evolution of critical situations deriving from the frequent repetition of specific movements. The lack of histological backup, which would have been impossible for ethical reasons, precludes our being able to state whether the increased local oxygenation was due to an increased number or caliber of the intra-tendon capillaries.

It is important to stress that an understanding of the pathogenesis of tissue adaptation to muscle-tendon overuse could provide a basis for setting up programs for the prevention of shoulder disorders in athletes practising overhead sports. In fact, the shoulder impingement syndrome is the second most frequent condition among sports overuse disorders, having an incidence of 8–10% in volleyball injuries [40,41]*.* Moreover, Verhagen et al. [70] report that shoulder overuse disorders account for the loss of an average of 6.5 weeks of training and/or competitions per year, being the main cause of forced suspension of sports activities.

Although the risk factors and etiopathogenesis of shoulder impingement in volleyball players have not yet been precisely defined, it seems reasonable that they should include overuse due to the specific movements these athletes commonly perform [71]*.* Our preliminary experience supports the utility of monitoring tendon neovascularization, as this is a possible parameter revealing the presence of a critical situation at risk of a disorder.

The strong points include the fact that tissue oximetry has a greater sensitivity in the study of perfusion of the microcirculation than other, previously used instrumental techniques such as color- and power-doppler [54].

## Conclusions

In conclusion, the finding of differences in oxygenation according to the role played in the volleyball team supports the hypothesis that variations in perfusion are determined by the different overuse conditions typical of each role. The use of this tool could allow certified athletic trainers, sports physicians and physiotherapists to monitor muscle-tendon overuse conditions *in itinere*, identifying and preventing the onset of disorders in subjects at higher risk. Clinical studies in larger case series with longer follow-up could help to define cut-off values for pathological as opposed to physiological tendon responses to the specific kinetic demands of overhead sports.

## Competing interests

The authors declare that they have no competing interests.

## Authors’ contributions

AN and BM drafted of the manuscript and reviewed the literature. AN, FF and DG conceived the study, participated in its coordination and in the acquisition of the data. PP was involved in analysis and interpretation of the correlation of oximetry results with Doppler images. LM and ST gave substantial contributions to statistical analysis and interpretation of data. All authors read and approved the final manuscript.

## Availability of supporting data

The data are deposited at University of Bari, Course of Motor and Sports Sciences (Gallone Donato’s thesis of degree).
